# Supplementary material for: COZOID: contact zone identifier for visual analysis of protein-protein interactions
Source: BMC Bioinformatics. 2018 Apr 6;19:125. doi: 10.1186/s12859-018-2113-6 (PMC5889581; doi:10.1186/s12859-018-2113-6)
Supplement: Supplementary file 2 — Software build. Executable binary file of the software tool. (ZIP 118,784 kb) [file 12859_2018_2113_MOESM2_ESM.zip › cozoid/java/docs/beansbinding-1.2.1-doc.zip!/index-files/index-3.html]

C-Index (Beans Binding)


---


|  |  |  |
| --- | --- | --- |
| **Overview** \| Package \| Class \| Use \| **Tree** \| **Deprecated** \| **Index** \| **Help** | |  |
| **PREV LETTER**   **NEXT LETTER** | **FRAMES**    **NO FRAMES**     **All Classes** |  |


A B C D E F G H I J K L M N O P Q R S T U V W 

---


## **C**

**changed(int)** - Method in class org.jdesktop.swingbinding.impl.ListBindingManager: **children** - Variable in class org.jdesktop.el.impl.parser.SimpleNode: **Choice()** - Method in class org.jdesktop.el.impl.parser.ELParser: **clearResolvedProperties()** - Method in class org.jdesktop.el.impl.lang.EvaluationContext: **coerce(Number)** - Method in class org.jdesktop.el.impl.lang.ELArithmetic.BigDecimalDelegate: **coerce(String)** - Method in class org.jdesktop.el.impl.lang.ELArithmetic.BigDecimalDelegate: **coerce(Number)** - Method in class org.jdesktop.el.impl.lang.ELArithmetic.BigIntegerDelegate: **coerce(String)** - Method in class org.jdesktop.el.impl.lang.ELArithmetic.BigIntegerDelegate: **coerce(Number)** - Method in class org.jdesktop.el.impl.lang.ELArithmetic: **coerce(Object)** - Method in class org.jdesktop.el.impl.lang.ELArithmetic: **coerce(String)** - Method in class org.jdesktop.el.impl.lang.ELArithmetic: **coerce(Number)** - Method in class org.jdesktop.el.impl.lang.ELArithmetic.DoubleDelegate: **coerce(String)** - Method in class org.jdesktop.el.impl.lang.ELArithmetic.DoubleDelegate: **coerce(Number)** - Method in class org.jdesktop.el.impl.lang.ELArithmetic.LongDelegate: **coerce(String)** - Method in class org.jdesktop.el.impl.lang.ELArithmetic.LongDelegate: **coerceToBoolean(Object)** - Static method in class org.jdesktop.el.impl.lang.ELSupport: **coerceToCharacter(Object)** - Static method in class org.jdesktop.el.impl.lang.ELSupport: **coerceToEnum(Object, Class)** - Static method in class org.jdesktop.el.impl.lang.ELSupport: **coerceToNumber(Object)** - Static method in class org.jdesktop.el.impl.lang.ELSupport: **coerceToNumber(Number, Class)** - Static method in class org.jdesktop.el.impl.lang.ELSupport: **coerceToNumber(Object, Class)** - Static method in class org.jdesktop.el.impl.lang.ELSupport: **coerceToNumber(String, Class)** - Static method in class org.jdesktop.el.impl.lang.ELSupport: **coerceToString(Object)** - Static method in class org.jdesktop.el.impl.lang.ELSupport: **coerceToType(Object, Class<?>)** - Method in class org.jdesktop.el.ExpressionFactory: Coerces an object to a specific type according to the EL type conversion rules. **coerceToType(Object, Class)** - Method in class org.jdesktop.el.impl.ExpressionFactoryImpl: **coerceToType(Object, Class)** - Static method in class org.jdesktop.el.impl.lang.ELSupport: **COLON** - Static variable in interface org.jdesktop.el.impl.parser.ELParserConstants: **column** - Variable in class org.jdesktop.el.impl.parser.SimpleCharStream: **columnCount()** - Method in class org.jdesktop.swingbinding.impl.ListBindingManager: **COMMA** - Static variable in interface org.jdesktop.el.impl.parser.ELParserConstants: **compare(Object, Object)** - Static method in class org.jdesktop.el.impl.lang.ELSupport: **Compare()** - Method in class org.jdesktop.el.impl.parser.ELParser: **CompositeELResolver** - Class in org.jdesktop.el: Maintains an ordered composite list of child `ELResolver`s. **CompositeELResolver()** - Constructor for class org.jdesktop.el.CompositeELResolver: **CompositeExpression()** - Method in class org.jdesktop.el.impl.parser.ELParser: **containsNulls(Object[])** - Static method in class org.jdesktop.el.impl.lang.ELSupport: **contextCreated(ELContextEvent)** - Method in interface org.jdesktop.el.ELContextListener: Invoked when a new `ELContext` has been created. **Converter**<S,T> - Class in org.jdesktop.beansbinding: `Converter` is responsible for converting a value from one type to another. **Converter()** - Constructor for class org.jdesktop.beansbinding.Converter: **convertForward(S)** - Method in class org.jdesktop.beansbinding.Converter: Converts a value from the source type to the target type. **convertReverse(T)** - Method in class org.jdesktop.beansbinding.Converter: Converts a value from the target type to the source type. **create(String)** - Static method in class org.jdesktop.beansbinding.BeanProperty: Creates an instance of `BeanProperty` for the given path. **create(Property<S, ?>, String)** - Static method in class org.jdesktop.beansbinding.BeanProperty: Creates an instance of `BeanProperty` for the given base property and path. **create(String)** - Static method in class org.jdesktop.beansbinding.ELProperty: Creates an instance of `ELProperty` for the given expression. **create(Property<S, ?>, String)** - Static method in class org.jdesktop.beansbinding.ELProperty: Creates an instance of `ELProperty` for the given base property and expression. **create()** - Static method in class org.jdesktop.beansbinding.ObjectProperty: Creates an instance of `ObjectProperty`. **create()** - Method in class org.jdesktop.el.impl.lang.FunctionMapperFactory: **create()** - Method in class org.jdesktop.el.impl.lang.VariableMapperFactory: **createAdapter(Object, String)** - Method in interface org.jdesktop.beansbinding.ext.BeanAdapterProvider: **createAdapter(Object, String)** - Method in class org.jdesktop.swingbinding.adapters.AbstractButtonAdapterProvider: **createAdapter(Object, String)** - Method in class org.jdesktop.swingbinding.adapters.JComboBoxAdapterProvider: **createAdapter(Object, String)** - Method in class org.jdesktop.swingbinding.adapters.JListAdapterProvider: **createAdapter(Object, String)** - Method in class org.jdesktop.swingbinding.adapters.JSliderAdapterProvider: **createAdapter(Object, String)** - Method in class org.jdesktop.swingbinding.adapters.JSpinnerAdapterProvider: **createAdapter(Object, String)** - Method in class org.jdesktop.swingbinding.adapters.JTableAdapterProvider: **createAdapter(Object, String)** - Method in class org.jdesktop.swingbinding.adapters.JTextComponentAdapterProvider: **createAutoBinding(AutoBinding.UpdateStrategy, SS, TS, Property<TS, TV>)** - Static method in class org.jdesktop.beansbinding.Bindings: Creates an instance of `AutoBinding` that binds a source object to a property of a target object. **createAutoBinding(AutoBinding.UpdateStrategy, SS, TS, Property<TS, TV>, String)** - Static method in class org.jdesktop.beansbinding.Bindings: Creates a named instance of `AutoBinding` that binds a source object to a property of a target object. **createAutoBinding(AutoBinding.UpdateStrategy, SS, Property<SS, SV>, TS, Property<TS, TV>)** - Static method in class org.jdesktop.beansbinding.Bindings: Creates an instance of `AutoBinding` that binds a property of a source object to a property of a target object. **createAutoBinding(AutoBinding.UpdateStrategy, SS, Property<SS, SV>, TS, Property<TS, TV>, String)** - Static method in class org.jdesktop.beansbinding.Bindings: Creates a named instance of `AutoBinding` that binds a property of a source object to a property of a target object. **createJComboBoxBinding(AutoBinding.UpdateStrategy, List<E>, JComboBox)** - Static method in class org.jdesktop.swingbinding.SwingBindings: Creates a `JComboBoxBinding` from direct references to a `List` and `JComboBox`. **createJComboBoxBinding(AutoBinding.UpdateStrategy, List<E>, JComboBox, String)** - Static method in class org.jdesktop.swingbinding.SwingBindings: Creates a named `JComboBoxBinding` from direct references to a `List` and `JComboBox`. **createJComboBoxBinding(AutoBinding.UpdateStrategy, SS, Property<SS, List<E>>, JComboBox)** - Static method in class org.jdesktop.swingbinding.SwingBindings: Creates a `JComboBoxBinding` from an object and property that resolves to a `List` and a direct reference to a `JComboBox`. **createJComboBoxBinding(AutoBinding.UpdateStrategy, SS, Property<SS, List<E>>, JComboBox, String)** - Static method in class org.jdesktop.swingbinding.SwingBindings: Creates a named `JComboBoxBinding` from an object and property that resolves to a `List` and a direct reference to a `JComboBox`. **createJComboBoxBinding(AutoBinding.UpdateStrategy, List<E>, TS, Property<TS, ? extends JComboBox>)** - Static method in class org.jdesktop.swingbinding.SwingBindings: Creates a `JComboBoxBinding` from a direct reference to a `List` and an object and property that resolves to a `JComboBox`. **createJComboBoxBinding(AutoBinding.UpdateStrategy, List<E>, TS, Property<TS, ? extends JComboBox>, String)** - Static method in class org.jdesktop.swingbinding.SwingBindings: Creates a named `JComboBoxBinding` from a direct reference to a `List` and an object and property that resolves to a `JComboBox`. **createJComboBoxBinding(AutoBinding.UpdateStrategy, SS, Property<SS, List<E>>, TS, Property<TS, ? extends JComboBox>)** - Static method in class org.jdesktop.swingbinding.SwingBindings: Creates a `JComboBoxBinding` from an object and property that resolves to a `List` and an object and property that resolves to a `JComboBox`. **createJComboBoxBinding(AutoBinding.UpdateStrategy, SS, Property<SS, List<E>>, TS, Property<TS, ? extends JComboBox>, String)** - Static method in class org.jdesktop.swingbinding.SwingBindings: Creates a named `JComboBoxBinding` from an object and property that resolves to a `List` and an object and property that resolves to a `JComboBox`. **createJListBinding(AutoBinding.UpdateStrategy, List<E>, JList)** - Static method in class org.jdesktop.swingbinding.SwingBindings: Creates a `JListBinding` from direct references to a `List` and `JList`. **createJListBinding(AutoBinding.UpdateStrategy, List<E>, JList, String)** - Static method in class org.jdesktop.swingbinding.SwingBindings: Creates a named `JListBinding` from direct references to a `List` and `JList`. **createJListBinding(AutoBinding.UpdateStrategy, SS, Property<SS, List<E>>, JList)** - Static method in class org.jdesktop.swingbinding.SwingBindings: Creates a `JListBinding` from an object and property that resolves to a `List` and a direct reference to a `JList`. **createJListBinding(AutoBinding.UpdateStrategy, SS, Property<SS, List<E>>, JList, String)** - Static method in class org.jdesktop.swingbinding.SwingBindings: Creates a named `JListBinding` from an object and property that resolves to a `List` and a direct reference to a `JList`. **createJListBinding(AutoBinding.UpdateStrategy, List<E>, TS, Property<TS, ? extends JList>)** - Static method in class org.jdesktop.swingbinding.SwingBindings: Creates a `JListBinding` from a direct reference to a `List` and an object and property that resolves to a `JList`. **createJListBinding(AutoBinding.UpdateStrategy, List<E>, TS, Property<TS, ? extends JList>, String)** - Static method in class org.jdesktop.swingbinding.SwingBindings: Creates a named `JListBinding` from a direct reference to a `List` and an object and property that resolves to a `JList`. **createJListBinding(AutoBinding.UpdateStrategy, SS, Property<SS, List<E>>, TS, Property<TS, ? extends JList>)** - Static method in class org.jdesktop.swingbinding.SwingBindings: Creates a `JListBinding` from an object and property that resolves to a `List` and an object and property that resolves to a `JList`. **createJListBinding(AutoBinding.UpdateStrategy, SS, Property<SS, List<E>>, TS, Property<TS, ? extends JList>, String)** - Static method in class org.jdesktop.swingbinding.SwingBindings: Creates a named `JListBinding` from an object and property that resolves to a `List` and an object and property that resolves to a `JList`. **createJTableBinding(AutoBinding.UpdateStrategy, List<E>, JTable)** - Static method in class org.jdesktop.swingbinding.SwingBindings: Creates a `JTableBinding` from direct references to a `List` and `JTable`. **createJTableBinding(AutoBinding.UpdateStrategy, List<E>, JTable, String)** - Static method in class org.jdesktop.swingbinding.SwingBindings: Creates a named `JTableBinding` from direct references to a `List` and `JTable`. **createJTableBinding(AutoBinding.UpdateStrategy, SS, Property<SS, List<E>>, JTable)** - Static method in class org.jdesktop.swingbinding.SwingBindings: Creates a `JTableBinding` from an object and property that resolves to a `List` and a direct reference to a `JTable`. **createJTableBinding(AutoBinding.UpdateStrategy, SS, Property<SS, List<E>>, JTable, String)** - Static method in class org.jdesktop.swingbinding.SwingBindings: Creates a named `JTableBinding` from an object and property that resolves to a `List` and a direct reference to a `JTable`. **createJTableBinding(AutoBinding.UpdateStrategy, List<E>, TS, Property<TS, ? extends JTable>)** - Static method in class org.jdesktop.swingbinding.SwingBindings: Creates a `JTableBinding` from a direct reference to a `List` and an object and property that resolves to a `JTable`. **createJTableBinding(AutoBinding.UpdateStrategy, List<E>, TS, Property<TS, ? extends JTable>, String)** - Static method in class org.jdesktop.swingbinding.SwingBindings: Creates a named `JTableBinding` from a direct reference to a `List` and an object and property that resolves to a `JTable`. **createJTableBinding(AutoBinding.UpdateStrategy, SS, Property<SS, List<E>>, TS, Property<TS, ? extends JTable>)** - Static method in class org.jdesktop.swingbinding.SwingBindings: Creates a `JTableBinding` from an object and property that resolves to a `List` and an object and property that resolves to a `JTable`. **createJTableBinding(AutoBinding.UpdateStrategy, SS, Property<SS, List<E>>, TS, Property<TS, ? extends JTable>, String)** - Static method in class org.jdesktop.swingbinding.SwingBindings: Creates a named `JTableBinding` from an object and property that resolves to a `List` and an object and property that resolves to a `JTable`. **createMethodExpression(ELContext, String, Class<?>, Class<?>[])** - Method in class org.jdesktop.el.ExpressionFactory: Parses an expression into a `MethodExpression` for later evaluation. **createMethodExpression(ELContext, String, Class, Class[])** - Method in class org.jdesktop.el.impl.ExpressionFactoryImpl: **createMethodExpression(Class, Class[])** - Method in class org.jdesktop.el.impl.lang.ExpressionBuilder: **createNode(String)** - Static method in class org.jdesktop.el.impl.lang.ExpressionBuilder: **createValueExpression(ELContext, String, Class<?>)** - Method in class org.jdesktop.el.ExpressionFactory: Parses an expression into a `ValueExpression` for later evaluation. **createValueExpression(Object, Class<?>)** - Method in class org.jdesktop.el.ExpressionFactory: Creates a ValueExpression that wraps an object instance. **createValueExpression(ELContext, String, Class)** - Method in class org.jdesktop.el.impl.ExpressionFactoryImpl: **createValueExpression(Object, Class)** - Method in class org.jdesktop.el.impl.ExpressionFactoryImpl: **createValueExpression(Class)** - Method in class org.jdesktop.el.impl.lang.ExpressionBuilder: **curChar** - Variable in class org.jdesktop.el.impl.parser.ELParserTokenManager: **currentToken** - Variable in exception org.jdesktop.el.impl.parser.ParseException: This is the last token that has been consumed successfully.

---


|  |  |  |
| --- | --- | --- |
| **Overview** \| Package \| Class \| Use \| **Tree** \| **Deprecated** \| **Index** \| **Help** | |  |
| **PREV LETTER**   **NEXT LETTER** | **FRAMES**    **NO FRAMES**     **All Classes** |  |


A B C D E F G H I J K L M N O P Q R S T U V W 

---
